# Failed upregulation of TFAM protein and mitochondrial DNA in oxidatively deficient fibers of chronic obstructive pulmonary disease locomotor muscle

**DOI:** 10.1186/s13395-016-0083-9

**Published:** 2016-02-18

**Authors:** Yana Konokhova, Sally Spendiff, R. Thomas Jagoe, Sudhakar Aare, Sophia Kapchinsky, Norah J. MacMillan, Paul Rozakis, Martin Picard, Mylène Aubertin-Leheudre, Charlotte H. Pion, Jean Bourbeau, Russell T. Hepple, Tanja Taivassalo

**Affiliations:** Department of Kinesiology, McGill University, 475 Pine Ave West, Room 222, Montreal, Quebec H2W1S4 Canada; Department of Critical Care Medicine, McGill University Health Center, Montreal, Canada; Departments of Oncology and Medicine, McGill University, Montreal, Canada; Division of Behavioral Medicine, Department of Psychiatry, Department of Neurology, and Columbia Translational Neuroscience Initiative, Columbia University College of Physicians and Surgeons, Columbia University Medical Center, New York, NY USA; Département de Kinanthropologie, Université du Québec à Montréal, Montreal, Canada; Respiratory Epidemiology and Clinical Research Unit, Center for Innovative Medicine (CIM), McGill University Health Centre, Montreal, Canada; Meakins Christie Laboratories, McGill University, Montreal, Canada

**Keywords:** Chronic obstructive pulmonary disease, Mitochondrial biogenesis, Mitochondrial DNA, mtDNA copy number, Muscle oxidative impairment, Oxidative stress, COX deficiency, TFAM, Laser capture microscopy

## Abstract

**Background:**

Low mitochondrial content and oxidative capacity are well-established features of locomotor muscle dysfunction, a prevalent and debilitating systemic occurrence in patients with chronic obstructive pulmonary disease (COPD). Although the exact cause is not firmly established, physical inactivity and oxidative stress are among the proposed underlying mechanisms. Here, we assess the impact of COPD pathophysiology on mitochondrial DNA (mtDNA) integrity, biogenesis, and cellular oxidative capacity in locomotor muscle of COPD patients and healthy controls. We hypothesized that the high oxidative stress environment of COPD muscle would yield a higher presence of deletion-containing mtDNA and oxidative-deficient fibers and impaired capacity for mitochondrial biogenesis.

**Methods:**

*Vastus lateralis* biopsies were analyzed from 29 COPD patients and 19 healthy age-matched controls for the presence of mtDNA deletions, levels of oxidatively damaged DNA, mtDNA copy number, and regulators of mitochondrial biogenesis as well the proportion of oxidative-deficient fibers (detected histologically as cytochrome *c* oxidase-deficient, succinate dehydrogenase positive (COX^−^/SDH^+^ )). Additionally, mtDNA copy number and mitochondrial transcription factor A (TFAM) content were measured in laser captured COX^−^SDH^+^ and normal single fibers of both COPD and controls.

**Results:**

Compared to controls, COPD muscle exhibited significantly higher levels of oxidatively damaged DNA (8-hydroxy-2-deoxyguanosine (8-OHdG) levels = 387 ± 41 vs. 258 ± 21 pg/mL) and higher prevalence of mtDNA deletions (74 vs. 15 % of subjects in each group), which was accompanied by a higher abundance of oxidative-deficient fibers (8.0 ± 2.1 vs. 1.5 ± 0.4 %). Interestingly, COPD patients with mtDNA deletions had higher levels of 8-OHdG (457 ± 46 pg/mL) and longer smoking history (66.3 ± 7.5 years) than patients without deletions (197 ± 29 pg/mL; 38.0 ± 7.3 years). Transcript levels of regulators of mitochondrial biogenesis and oxidative metabolism were upregulated in COPD compared to controls. However, single fiber analyses of COX^−^/SDH^+^ and normal fibers exposed an impairment in mitochondrial biogenesis in COPD; in healthy controls, we detected a marked upregulation of mtDNA copy number and TFAM protein in COX^−^/SDH^+^ compared to normal fibers, reflecting the expected compensatory attempt by the oxidative-deficient cells to increase energy levels; in contrast, they were similar between COX^−^/SDH^+^ and normal fibers in COPD patients. Taken together, these findings suggest that although the signaling factors regulating mitochondrial biogenesis are increased in COPD muscle, impairment in the translation of these signals prevents the restoration of normal oxidative capacity.

**Conclusions:**

Single fiber analyses provide the first substantive evidence that low muscle oxidative capacity in COPD cannot be explained by physical inactivity alone and is likely driven by the disease pathophysiology.

## Background

Locomotor muscle dysfunction is a disabling, extra-pulmonary occurrence in chronic obstructive pulmonary disease (COPD) and a major independent contributory factor in the severe exercise intolerance experienced by patients [1, 2]. Low muscle oxidative capacity is a well-established aspect of this dysfunction, playing a major role in the poor muscle endurance and high fatigability that is typical of COPD patients [3–6]. The pathophysiological mechanisms that underlie the loss of muscle oxidative capacity in COPD are unclear and likely relate to an interplay between muscle disuse and other disease-specific contributors including inflammation, hypoxia, and oxidative stress. A precise understanding of contributing mechanisms is important because patients who develop locomotor muscle dysfunction are heavier users of health-care resources and have shorter life expectancies compared to those with the same pulmonary disease severity with normal muscle function [7].

Skeletal muscle oxidative capacity is highly dependent on the quantity of functional mitochondria, which can vary in response to physiological cues such as physical in/activity, disease, and aging [8–12]. In COPD locomotor muscle, reductions in both mitochondrial density and enzyme activities have been established [3, 4, 11], and more recently, low mitochondrial DNA (mtDNA) copy number has been reported [13]. These findings are consistent with a reduction in mitochondrial biogenesis, where levels of the key regulatory factor peroxisome proliferator-activated receptor *γ* coactivator 1α (PGC-1α) have been shown to be lower in COPD compared to healthy locomotor muscle [5, 14]. Furthermore, protein levels of its downstream effector, mitochondrial transcription factor A (TFAM), have also been shown to be reduced in COPD locomotor muscle [14]. TFAM plays a critical role in the transcription and replication of mtDNA, both of which are necessary processes for the synthesis of functional mitochondria and maintenance of mtDNA copy number [15, 16]. Taken together, current evidence suggests that disturbed expression of mitochondrial biogenic regulatory factors may underlie the reduction in skeletal muscle oxidative capacity in COPD [14, 17]. However, whether these alterations reflect the normal response of muscle to reduced contractile activity associated with the patients’ very sedentary lifestyle [18, 19] or pathologically impaired mitochondrial biogenesis secondary to the COPD systemic milieu of inflammation and high oxidative stress is currently not established.

In considering potential pathophysiological mechanisms that might compromise mitochondrial biogenesis responses, high oxidative stress is well-characterized in COPD locomotor muscle [13, 20, 21]. In fact, muscle oxidative stress has been associated with a reduced endurance capacity of the quadriceps muscle itself in severe COPD [22], and more recently, inverse correlations between oxidative stress and markers of muscle oxidative capacity have been reported in more mild COPD patients [5]. Interestingly, although there is some evidence for oxidative damage to mtDNA in COPD locomotor muscle [13], the cellular functional consequences of this for skeletal muscle oxidative capacity have not been considered. As first established in patients with mitochondrial disease [23], and subsequently observed in healthy aging muscle [24], accumulation of mtDNA mutations relative to wild-type mtDNA within a muscle fiber can impair the synthesis of functional electron transport chain complexes. The resulting cellular oxidative enzyme deficiency can be detected histochemically as cytochrome *c* oxidase-deficient, succinate dehydrogenase positive (COX^−^/SDH^+^) fibers. These fibers typically exist alongside oxidative-normal fibers within the same muscle and occur normally in low abundance in healthy aged muscle and in much higher abundance in patients with primary mitochondrial disease [25, 26]. Interestingly, single fiber observations of mitochondrial biogenesis within these COX^−^/SDH^+^ fibers have revealed an upregulation of mtDNA copy number and TFAM protein levels [27–29] compared to oxidative-normal fibers, and at the whole muscle level, this is associated with activation of the mitochondrial biogenesis signaling cascade [30], reflecting an attempted compensatory response to the cellular oxidative deficiency, noting that this response at the single fiber level occurs despite the very sedentary behavior typical of patients with mitochondrial disease [31]. Thus, comparison of the mitochondrial biogenesis response between normal and COX^−^/SDH^+^ fibers could reveal whether the reduced muscle oxidative capacity in COPD is impaired beyond that explainable by the very low physical activity levels of these patients.

On the basis of the above, the objectives of our study were to evaluate in locomotor muscle of moderate-to-severe COPD patients and healthy age-matched individuals the level of oxidative stress (4-hydroxynonenal levels for protein oxidation and 8-hydroxyguanosine for oxidative DNA damage), mtDNA integrity (mtDNA deletions and copy number), and the corresponding regulators of mtDNA copy number and mitochondrial biogenesis (PGC-1α, PGC-1β, NRF1, and TFAM levels) and oxidative metabolism (peroxisome proliferator-activated receptors (PPARs)) in whole muscle homogenate; and at the single fiber level, the proportion of COX^−^/SDH^+^ fibers and differences in mtDNA copy number and TFAM content between oxidatively impaired versus normal muscle fibers. We hypothesized that COPD muscle would exhibit elevated levels of oxidatively damaged DNA and altered mtDNA integrity reflected as a higher presence of deletion-containing mtDNA and COX^−^/SDH^+^ fibers compared to healthy muscle. Furthermore, we hypothesized that at the single fiber level, there would be a compromised mitochondrial biogenesis response in the oxidative-deficient fibers compared to healthy age-matched controls. The significance of this latter hypothesis is that if confirmed, it would demonstrate for the first time convincing evidence that the low muscle oxidative capacity in COPD muscle is not merely a consequence of their sedentary lifestyle and instead implicate a myocellular pathology in this response.

Here, we show that COPD muscle contains a higher abundance of oxidatively deficient fibers and alterations in mtDNA integrity. Furthermore, using techniques appropriate for single fiber studies, we have demonstrated a failed upregulation of both mtDNA copy number and the upstream regulator TFAM protein in these oxidatively deficient fibers, revealing an impairment in mitochondrial biogenesis. Some of the results of these studies have been previously reported in abstract form [32, 33].

## Methods

### Study participants

This study was approved by the Institutional Review Board for human studies of the Montreal Chest Institute (Montréal, QC, Canada). Twenty-nine ambulatory male patients with moderate-to-severe COPD based on the Global Initiative for Chronic Obstructive Lung Disease classification of disease severity [34] were recruited from the outpatient COPD clinic of the Montreal Chest Hospital of the McGill University Health Center. Patients were excluded if: (1) they reported an exacerbation requiring the use of corticosteroids or antibiotics within the preceding month; (2) they were on long-term oxygen therapy; (3) they used oral corticosteroids; (4) they participated in a pulmonary rehabilitation program within the preceding year; and (5) they had a known comorbidity that could interfere with our outcomes measures, including severe cardiac, diabetes, neurologic, neuromuscular, cancer, and/or orthopedic disease/condition. Nineteen healthy male age-matched sedentary control subjects were recruited through word of mouth and local advertisements (Table 1). After obtaining written informed consent for procedures and publishing, all participants underwent clinical and physiological evaluation and, in a separate visit, a biopsy of the mid-portion of the *vastus lateralis* muscle using the modified Bergstrom needle method with suction [35]. Tissue for genetic analysis was snap frozen in liquid nitrogen, while muscle histology samples were mounted transversely using tragacanth gum and frozen in liquid nitrogen-precooled isopentane and stored at −80 °C.

**Table 1:** Descriptive characteristics of healthy controls and patients with COPD

Characteristics	Control (*n* = 19)	COPD (*n* = 29)
Age, years	68 ± 6	66 ± 5
Smoking, pack-years	3.4 ± 10.4	64.4 ± 37.1*
Weight, kg	80.1 ± 14.2	71.2 ± 17.2
Height, m	171.9 ± 4.4	169.7 ± 7.5
BMI, kg/m^2^	26.8 ± 3.8	28.8 ± 11.9
FEV_1_, % pred	107.9 ± 21.9	40.7 ± 13.9*
FEV_1_/FVC, % pred	78.5 ± 7.3	51.4 ± 13.9*
TLC, % pred	103.1 ± 13.3	122.2 ± 17.2*
RV, % pred	95.8 ± 37.1	172.1 ± 37.4*
DLCO, % pred	105.3 ± 14.3	53.2 ± 16.7*
Peak VO_2_, % pred	92.2 ± 17.4	38.4 ± 13.3*
Peak work, % pred	112.3 ± 21.6	36.5 ± 19.3*
Fiber CSA (μm^2^)	5684.6 ± 1328.7	5007.9 ± 1181.4
Fiber type 1, %	48.10 ± 20.3	24.0 ± 9.8*
Type II shifters^a^, %	20	73*

Values presented as mean ± SD

*Abbreviations: COPD* chronic obstructive pulmonary disease, *BMI* body mass index, *FEV*_*1*_ forced expiratory volume in one second , *FVC* forced vital capacity, *TLC* total lung capacity, *RV* residual volume, *DLCO* lung diffusion capacity, *CSA* cross section area

*p < 0.05

^a^Number of patients with <27 % myosin heavy chain type 1 fibers

### Clinical and physiological evaluation

Lifetime tobacco exposure was calculated for each participant, as pack-year units, by multiplying the number of cigarettes smoked per day by the number of years smoked and dividing by 20 (*20 cigarettes per pack) [36]. Height in meters and weight in kilograms were assessed on a standard scale. Pulmonary function testing included spirometry and whole-body plethysmography to measure forced expiratory volume (FEV_1_), residual volume, total lung capacity, and diffusing capacity of the lung for carbon monoxide. All values obtained were expressed as a percentage of reference values. A symptom-limited incremental peak exercise test was performed on an electromagnetically braked upright cycle ergometer (Ergoline 800®) according to established guidelines [37] to determine peak work capacity and peak rate of oxygen consumption (VO_2_). Throughout this test, electrocardiogram, ventilatory parameters, and arterial oxygen saturation through finger oximetry were continuously monitored. Values obtained were expressed as a percentage of predicted as per Blackie et al. [36].

### Quantification of DNA damage

DNA was extracted from muscle samples using a QIAgen DNeasy Blood and Tissue Kit (QIAGEN, Valencia, CA) according to the manufacturer’s instructions and concentrated to 20 mg/ml. Digest mix was prepared by adding 250 units Benzonase, 300 m units phosphodiesterase I (Sigma-Aldrich, P-3243), and 200 units alkaline phosphatase (Sigma-Aldrich, P-7923) to 5 mL Tris-HCl buffer (20 mM, pH 7.9) containing 100 mM NaCl and 20 mM MgCl_2_ (24). DNA samples (3 μg) in digest mix were incubated at 37 °C for 6 h [38].

#### 8-OHdG assay

Levels of oxidatively damaged DNA, 8-hydroxy-2-deoxyguanosine (8-OHdG), the nucleoside’s most sensitive to modification by oxidative stress [39–41], were measured using a competitive ELISA kit (DNA/RNA Oxidative Damage EIA Kit, Cayman Chemicals, Ann Arbor, Michigan) following the manufacturer’s instructions. Samples were run in triplicates, along with a negative control and quantified using an eight-point standard curve (10–3000 pg/ml). The concentration of 8-OHdG (pg/ul) was standardized per total DNA content (1 μg per well).

### Detection of mtDNA deletions in muscle homogenate samples

Long-range PCR was performed to amplify a 10,774-bp fragment of the major arc region, where the majority of mtDNA deletions occur [42]. Reactions consisted of 1 μl of DNA template, 5 μl reaction buffer, 8.75 μl 0.35 mM dNTPs, 0.7 μl Expand Taq (TaKaRa), 20 uM of both forward and reverse primers 10 pmol/μl (primers: forward 5′-AGA TTT ACA GTC CAA TGC TTC-3′, reverse 5′-AGA TAC TGC GAC ATA GGG TG-3′), and 21.55 μl dH_2_O. Amplification was performed on a thermal cycler under the following conditions: 3 min at 93 °C; 30 cycles of 93 °C for 30 s, 58 °C for 30 s, and 68 °C for 12 min; with an additional 5 s for every cycle and then a final extension of 11 min at 68 °C. Amplified PCR products (consisting of full-length 10,774-bp amplicons along with smaller amplicons with a mtDNA deletion) were visualized using gel electrophoresis on a 0.7 % agarose gel stained with SYBR Safe (Life Technologies). Fifteen microliters of the PCR product was loaded along with 0.5 μl of loading dye, and a 1 kb + ladder (Invitrogen) was loaded to enable estimation of the product size. The gel was run for 150 min at 80 V, and bands were visualized on a UV gel documentation system G: BOX (Syngene).

### Myocellular oxidative capacity

Dual COX and SDH histochemistry is a technique routinely employed to detect in situ myofibers with a respiratory chain deficiency due to a high presence of mtDNA mutations [43, 44]. Since mtDNA encodes three subunits of complex IV (COX), and none of complex II (SDH), mtDNA deletions often result in low COX activity, while SDH activity remains unaffected [26]. As such, fibers with normal COX activity (COX^+^/SDH^+^) will demonstrate a brown reaction product (Fig. 2a, left panel), whereas fibers with reduced or absent COX activity secondary to mtDNA defects will only stain for the SDH blue reaction product (COX^−^/SDH^+^) (Fig. 2a, mid panel).

Ten-micrometer muscle sections from 21 COPD and 14 control subjects were first incubated in COX medium (100 μM cytochrome *c*, 4 mM diaminobenzidine tetrahydrochloride (DAB), and 2 % *w*/*v* catalase in 0.2 M phosphate buffer, pH 7.0) at 37 °C for 45 min. Sections were then washed in PBS and incubated in SDH medium for CII activity (130 mM sodium succinate, 200 μM phenazine methosulphate, 1 mM sodium azide, 1.5 mM nitroblue tetrazolium in 0.2 M phosphate buffer, pH 7.0) at 37 °C for 45 min. Sections were washed in PBS, pH 7.4, dehydrated in a graded ethanol series (70, 95, 2 × 100 %), cleared in Xylene (Sigma-Aldrich, Saint Louis, MO, USA), and mounted in DPX (Sigma-Aldrich, Saint-Louis, MO, USA).

Slides were imaged on a Scanscope XT digital slide scanner (Aperio, Vista, CA, USA) and analyzed using ImageJ software (http://imagej.nih.gov/ij/, National Institutes of Health, Bethesda, MD). The total number of COX^+^/SDH^+^, COX^−^/SDH^+^, and COX^−^/SDH^−^ fibers (fibers with reduced activity of both COX and SDH) were counted using ImageJ (National Institutes of Health), for the entire muscle section (average of 366 fibers per section) in each subject by an observer blinded to the group condition, with a subset being counted by a second blinded observer to confirm classification.

### Quantification of mtDNA copy number in muscle homogenate and single fibers

MtDNA copy number was quantified using a standard curve of the mitochondrial encoded NADH dehydrogenase 1 region (MTND1) [45] in both homogenate muscle and laser captured single COX-normal and COX-deficient muscle fibers. This region of the mitochondrial genome is in the minor arc and is therefore preserved in 95 % of cases of mtDNA deletions [42]. Samples were run on a MTND1 TaqMan® qPCR assay with mtDNA copy number being determined relative to the standard curve, and then normalized to the total area of the captured cells.

The standard curve containing the MTND1 region was generated from serial dilutions of template mtDNA (1020-bp fragment, forward primer: 5′-TGT AAA ACG ACG GCC AGT CAG CCG CTA TTA AAG GTT CG 3′, reverse 5′-CAG GAA ACA GCT ATG ACC AGA GTG CGT CAT ATG TTG TTC-3′). The products were then separated on a 1 % agarose gel, and the mtDNA fragment was then extracted using a QiAquick Gel Extraction Kit and quantified using a NanoDrop-2000 spectrophotometer (Thermo Scientific, USA).

Groups of approximately 100 fibers per subject were laser captured and used to determine values for the muscle homogenate measurements with the area captured being noted. For single fiber analysis, individual COX-normal and COX-deficient fibers (where present) were laser captured and analyzed for mtDNA copy number. Laser capture was performed using an ArcturusXT™ LCM (Thermo Fisher Scientific) and Arcturus® CapSure® Macro LCM caps, with identified cells being traced and then lasered out from a pen membrane slide stained only for SDH in order not to disrupt the qPCR protocol [46]. DNA was then extracted from the cells using 20 μl of lysis buffer (1 % Tween, 20 μg/μl proteinase K in 50 mM Tris HCl) and incubating at 55 °C for 4 h and 95 °C for 10 min. The MTND1 gene was amplified (forward primer: 5′-CCC TAA AAC CCG CCA CAT CT-3′ and reverse 5′-GAG CGA TGG TGA GAG CTA AGG T-3′) under the following cycle conditions: 2 min at 50 °C, 10 min at 95 °C, followed by 40 cycles of 15 s at 95 °C and 60 s at 60 °C. Only samples with threshold cycle (CT) values 3 cycles away from the no template control were considered usable

### Quantification of transcripts involved in oxidative metabolism and mitochondrial biogenesis

Total RNA was extracted from samples using the RNeasy Lipid Tissue Mini Kit (Qiagen), according to manufacturer’s instructions. RNA concentration and purity (*A*260/*A*280 ratios >1.8) were assessed using a spectrophotometer. RNA (1 μg) was reverse transcribed to cDNA using qScript™ cDNA Synthesis Kit (Quanta BioSciences) according to the manufacturer’s instructions. Primers for TFAM (forward TGCAGGAGAAAAAGCCCTAA, reverse GCATTTGTCCCGAGATGTTT (NCBI ref: NM_001270782.1)), PPARGC1alpha (PGC-1α) (forward TTTCCTTTTGCCATGGAATC, reverse: GAAAGAACCGCTGAACAAGC (NCBI ref: XM_005248130.1)), PPAR gamma (PPARγ) (forward GACCACTCCCACTCCTTTGA, reverse GATGCAGGCTCCACTTTGAT (NCBI ref: NM_005037.5)), PPAR delta (PPARδ) (forward CCACTGACCCAACTGATCCT, reverse GAGGGAACCCTGCCTACTTC (NCBI ref: NM_001171818.1)), PPARGC1 beta (PGC-1β) (forward ACTATCTCGCTGACACGCAG, reverse GGCTGTACTGGTTGGGTTCA (NCBI ref: XM_011537553.1)), NRF1 (forward CACAGAAAAGGTGCTCAAAGGAT, reverse GGCCACTGCATGTGCTTCTA (NCBI ref: NM_001040110.1)) were designed with Primer 3 plus. TATA box binding protein (TBP) (primers: forward TATAATCCCAAGCGGTTTGC-3, reverse GCTGGAAAACCCAACTTCTG (NCBI ref: NM_001172085.1)) were used as a reference gene) was used to quantify the mRNA. The cDNA was amplified using Power SYBR® Green PCR Master Mix (Life Technologies) under the following conditions: 95 °C for 10 min followed by 40 cycles of 95 °C for 15 s and 55 °C for 60 s. All real-time PCR experiments were performed in triplicates along with melt curve analysis to assess primer dimer formation or contamination. Then, the comparative CT method was used to calculate fold changes in expression in the COPD groups compared with the control groups, where ΔCT = CT of gene of interest − CT of TBP and ΔΔCT = ΔCT of control group genes − ΔCT of COPD group genes.

### Western blot analysis

TFAM and 4-hydroxynonenal (HNE) quantification was performed by SDS-PAGE and Western blot. Between 10 and 55 mg of muscle was homogenized with 10 volumes of extraction buffer (50 mM Tris base, 150 mM NaCl, 1 % Triton X-100, 0.5 % sodium deoxycolate, 0.1 % sodium dodecyl sulfate (SDS), and 10 μl/ml of protease inhibitor cocktail (Sigma-Aldrich)) using a MM400 robot homogeniser (Retsch) then, incubated at 4 °C for 2 h (shaking) and centrifuged at 12,000*g* for 20 min (4 °C). The supernatant was removed, and the protein content was assessed using the Bradford technique. Protein samples were diluted in extraction buffer and Laemli buffer to a final concentration of 2 μg/μl before boiling at 95 °C for 5 min.

Fifteen micrograms of protein was loaded onto a 12 % acrylamide gel for electrophoresis and then transferred to polyvinylidene fluoride membranes (Life Sciences). Membranes were then blocked in 5 % (*w*/*v*) semi-skimmed milk for 1 h at room temperature and probed overnight at 4 °C with mitochondrial transcription factor A (TFAM) (Abcam) or HNE (anti-4-hydroxynonenal antibody) in 5 % BSA. Rabbit polyclonal anti β-tubulin (1:500) (Abcam) was used as a loading control for TFAM analysis. Membranes were subsequently incubated with horseradish peroxidase (HRP)-conjugated secondary antibody (Abcam; 5 % milk) for 1 h at room temperature. Protein bands were detected using SuperSignal™ West Pico Chemiluminescent Substrate (Thermo Scientific) and imaged with a G-Box Chem imaging system.

### Immunofluorescence labeling

#### TFAM

Muscle sections were first fixed in 4 % paraformaldehyde for 30 min. Antigen retrieval was performed by submerging sections into boiling EDTA (1 mM, pH 8.0) for 1 min and the sections were then permeabalized with 0.1 % Triton for 15 min at room temperature. Samples were then blocked in 10 % normal goat serum (Life Technologies) for 30 min, probed for 2 h with TFAM (Abcam 1:150 dilution, room temperature). Following three washes with PBS, sections were incubated with Alexa Fluor 488 IgG for 1 h, again at room temperature, washed in PBS and mounted in ProLong gold (Life Technologies).

#### Myosin heavy chain

Muscle sections were blocked in 10 % normal goat serum and incubated with the following primary antibodies (all from Developmental Studies Hybridoma Bank; diluted in blocking serum) for 1 h at room temperature: BA-F8 (mouse anti-myosin heavy chain (MHC)1 IgG2b, 1:25), Sc-71 (mouse anti-MHC2a IgG1, 1:200), and 6H1 (mouse anti-MHC2x IgM, 1:25). Following three washes in PBS, sections were probed with the following secondary antibodies: Alexa Fluor 350 IgG2b (y2b) goat anti-mouse 1:500, Alexa Fluor 594 IgG1 (y1) goat anti-mouse 1:100, Alexa Fluor 488 IgM goat anti-mouse 1:500, and Alexa Fluor 488 IgG goat anti-rabbit 1:500. Sections were then washed in PCS and mounted using ProLong gold. Images were captured on a Zeiss Axio Imager M2 fluorescent microscope (Zeiss, Germany).

### Statistical analyses

Statistical analysis was performed using GraphPad Prism (GraphPad Software Inc.). Results are presented as mean ± SEM, with significance set at *P* < 0.05. Data was tested for normality using the Pearson omnibus normality test. Values more than two standard deviations away from the mean were identified as outliers. In some cases, it was not possible to perform all investigations on all subjects due to tissue availability. Where this has occurred, details are provided in the figure legends.

Two-way ANOVAs were used to compare mtDNA copy number as well as TFAM protein content in single COX^−^/SDH^+^ and COX^+^/SDH^+^ fibers between the COPD and control groups, with Bonferroni’s post hoc tests for multiple comparisons. The chi-square test was used on raw counts to analyze differences in proportion of COX^+^/SDH^+^, COX^-^/SDH^+^, and COX^low^/SDH^low^ between the COPD and control groups. Two group comparisons were assessed using *t* test or Mann-Whitney test depending on distribution, with all tests being performed two-tailed.

## Results

### Participant characteristics

Characteristics of the study participants, all elderly males, are presented in Table 1. As seen by the pulmonary function testing, the COPD group had severe airflow obstruction. Compared to the healthy controls, COPD patients had a significantly higher smoking pack history and lower predicted peak aerobic and work capacity. In addition, COPD patient muscle displayed a low oxidative phenotype as reflected by a significantly lower type 1 fiber composition compared to the controls.

### Higher prevalence of oxidative damage and mtDNA deletions in COPD muscle

**Fig. 1:**
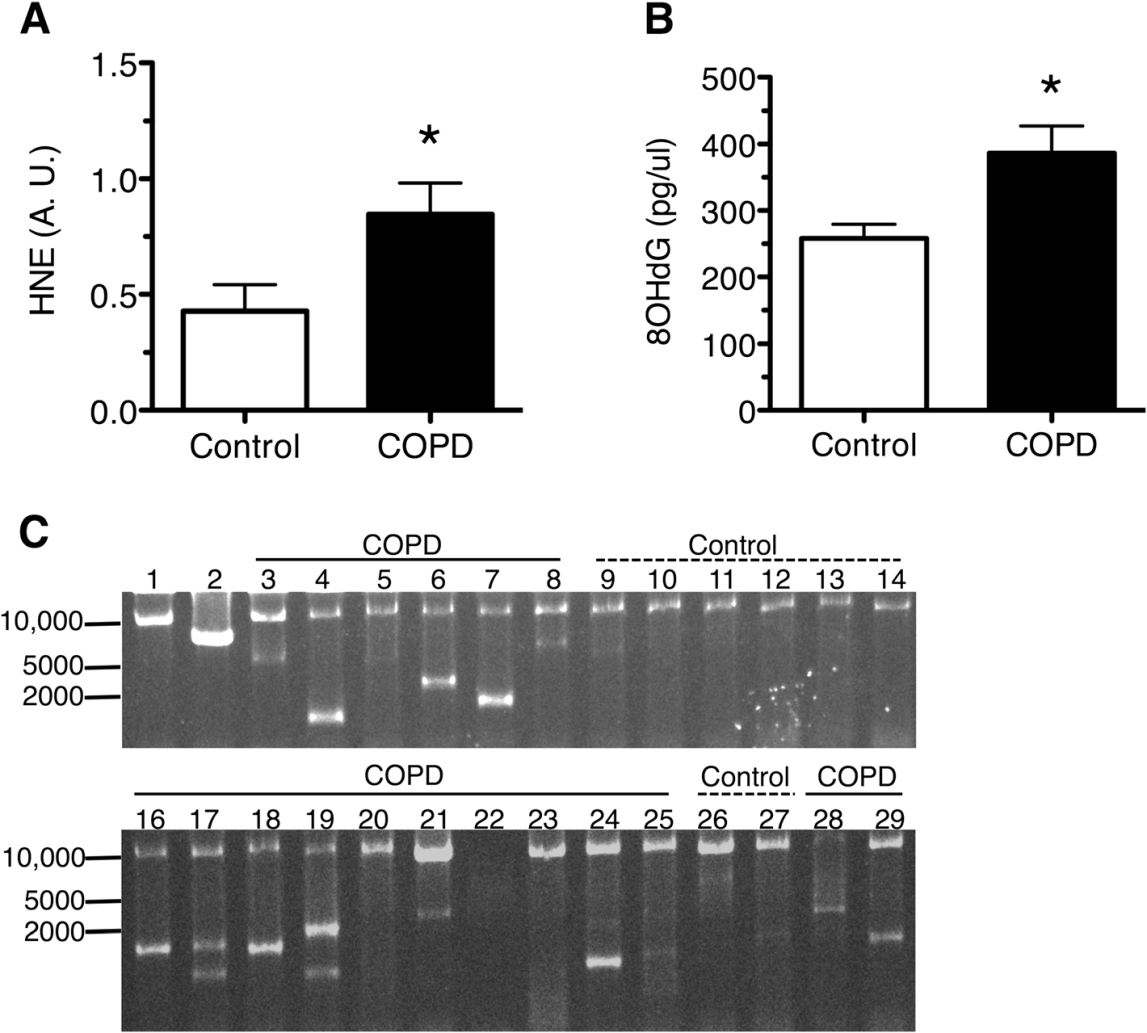
Higher prevalence of damaged protein and DNA in COPD *vastus lateralis* skeletal muscle. **a** Higher levels of 4-hydroxynonenal (HNE)-adduct containing proteins in COPD patients (0.85 ± 0.13; *n* = 17) compared to age-matched controls (0.43 ± 0.11; *n* = 10; *P < 0.05). **b** Higher concentration of oxidatively damaged guanosine (pg of 8-OHdG per uL of total DNA) in COPD muscle (386 ± 40.64; *n* = 29) compared to age-matched controls (258.0 ± 21.44; *n* = 16; *P < 0.01). **c** Representative image of long-range PCR gel demonstrating mtDNA deletions in COPD and control subjects. *Lane 1*: wild-type mtDNA taken from a young adult demonstrating the full-length (10.774 kb) amplified PCR product. *Lane 2*: mtDNA taken from a subject known to harbor a single mtDNA deletion resulting in an amplified product of 7.1 kb, thus demonstrating the specificity of this technique in detecting mtDNA deletions. *Lanes 3–8, 16–25, and 28–29* show the presence of deletion-containing amplicons together with wild-type mtDNA product in most COPD subjects. *Lanes 9–14 and 26–27* show the presence of the full-length wild-type mtDNA product and deletion-containing amplicons in control subjects. Graphs show mean ± SEM

The marker of oxidatively damaged proteins, 4-hydroxynonenal (HNE) adduct (Fig. 1a), was twofold higher in the *vastus lateralis* muscle of COPD patients (0.85 ± 0.13; *n* = 17) compared to age-matched controls (0.43 ± 0.11; *n* = 10; *P* < 0.05; Fig. 1a). The concentration of oxidatively damaged guanosine base (8-OHdG) was 50 % higher in COPD patients (mean 387 ± 40 pg/μL) compared to control subjects (mean 258 ± 21 pg/μL; *P* < 0.04; Fig. 1b). Furthermore, these differences in oxidative damage were associated with a higher frequency of COPD patients exhibiting mtDNA deletions in whole muscle homogenates (Fig. 1c), with 21 of 29 patients (72 %) versus only 3 out of 19 controls (16 %) harboring mtDNA deletions *P * < 0.001).

**Fig. 2:**
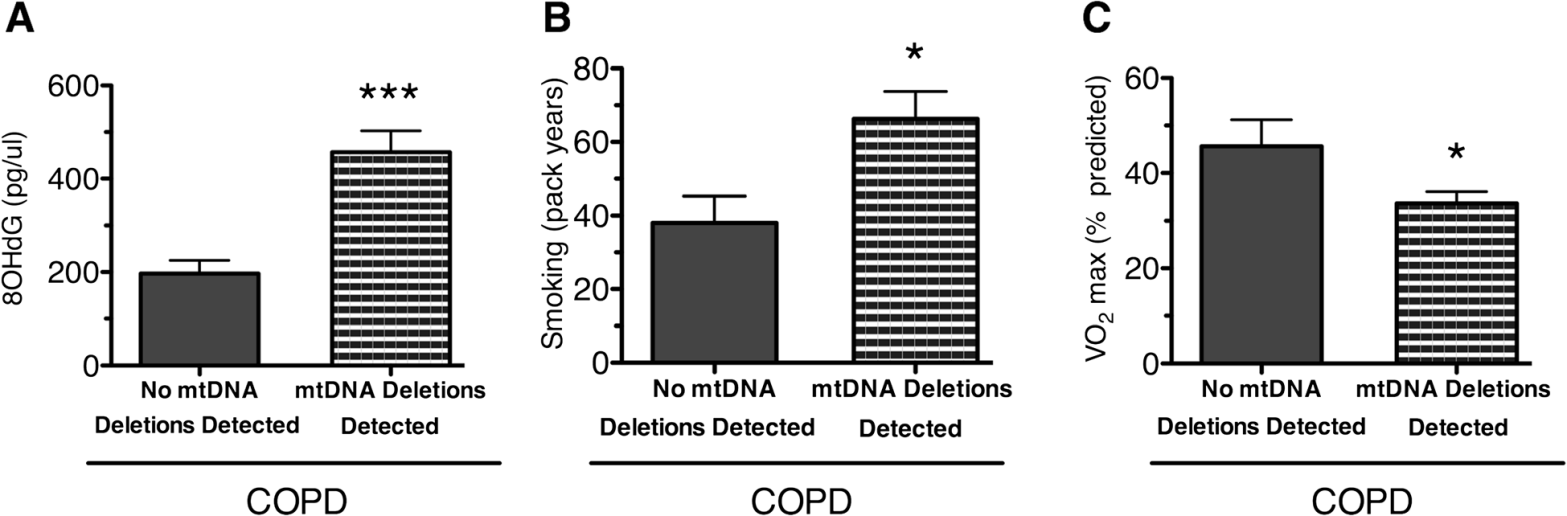
Presence of mtDNA deletions in COPD locomotor muscle corresponds to higher levels of DNA damage, smoking history, and aerobic capacity. **a** Higher levels of oxidatively damaged guanosine (pg of 8-OHdG per mL of total DNA) in COPD patients with mtDNA deletions (456.8 ± 45.7) compared to patients without detectable mtDNA deletions (196.9 ± 28.6; ***P < 0.001). **b** Higher number of smoking pack-years in COPD patients harboring mtDNA deletions (66.3 ± 7.5) compared to COPD patients without detectable mtDNA deletions (38.0 ± 7.3). **c** Lower maximal oxygen consumption in COPD patients harboring mtDNA deletions (33.7 ± 2.4 % predicted) compared to COPD patients without detectable mtDNA deletions (45.6 ± 5.6 %). Graphs show mean ± SEM (*P < 0.05)

Consistent with oxidative damage being an important driver of the exacerbation of mtDNA deletion in COPD muscle, COPD patients exhibiting mtDNA deletions had higher levels of 8-OHdG (*n* = 21; mean 457 ± 46 pg/μL) compared to COPD patients *without* mtDNA deletions (*n* = 8; mean 197 ± 28 pg/μL, *P* < 0.002; Fig. 2a). Furthermore, COPD patients harboring mtDNA deletions had a longer smoking history as measured by pack-years (COPD patients with mtDNA deletions, 66.3 ± 7.5 years; with no mtDNA deletions, 38.0 ± 7.3 years; *P* < 0.05) (Fig. 2b). Interestingly, COPD patients harboring mtDNA deletions had a lower maximal oxygen consumption than those without deletions (patients with mtDNA deletions, 33.7 ± 2.4 % predicted maximal VO_2_; patients with no mtDNA deletions, 45.6 ± 5.6 % predicted maximal VO_2_; Fig. 2c).

### COX-deficient fibers are more prevalent in COPD locomotor muscle

**Fig. 3:**
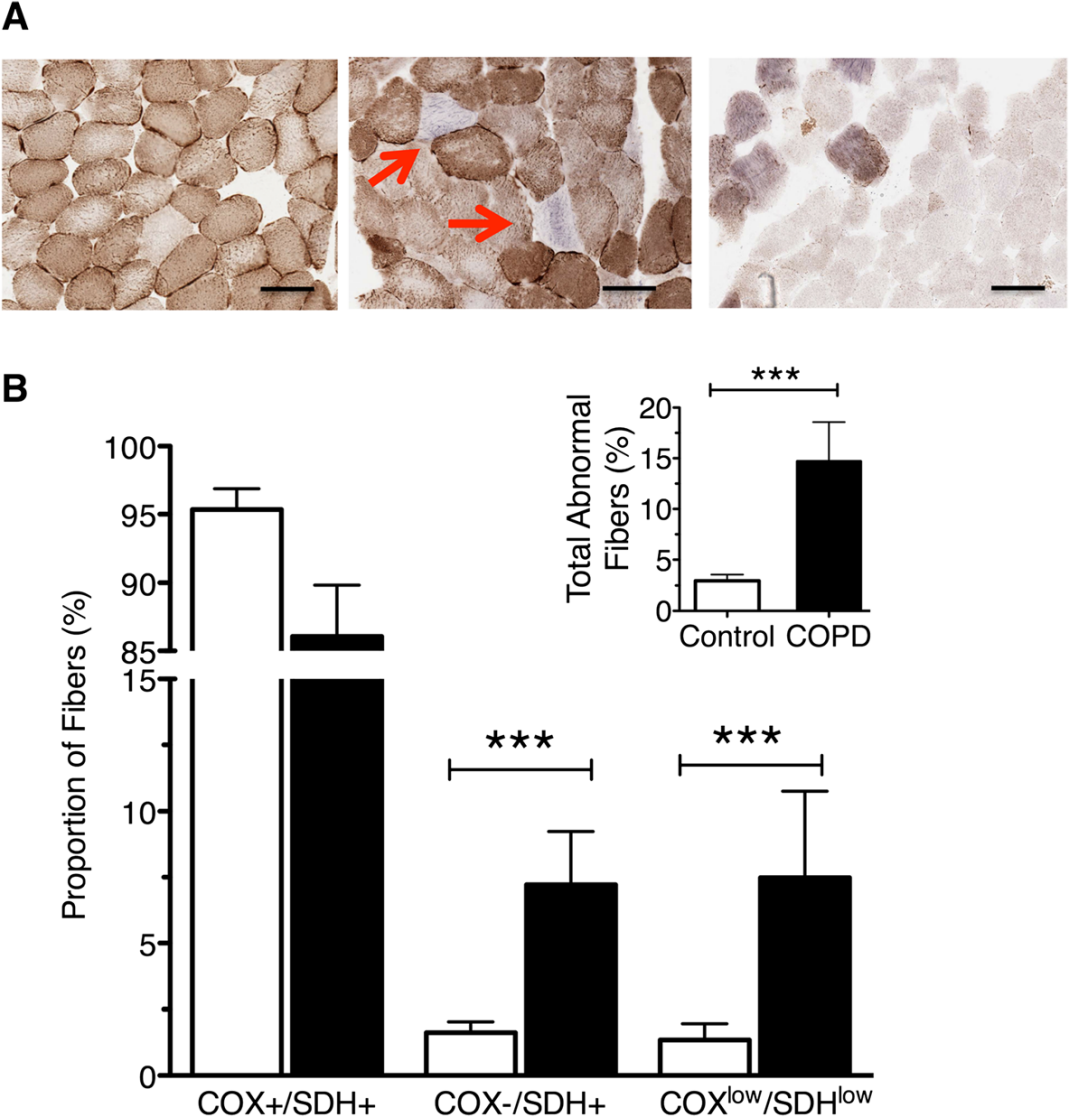
Dual COX/SDH histological staining to detect respiratory chain deficiency attributable to mtDNA deletions in *vastus lateralis* muscle. **a** Cross-sectional images from a representative control subject demonstrating uniform, normal-COX staining (*left*) and a COPD patient with respiratory-deficient fibers highlighted (*red arrows*, *middle*) as well as the lack of staining (*right*) reflecting depletion of mitochondria in muscle fibers. **b** Proportions of COX-normal (COX^+^/SDH^+^), COX-deficient (COX^−^/SDH^+^), and mitochondria-depleted (COX^low^/SDH^low^) fibers presented as individual percentages in controls (*n* = 14) and COPD subjects (*n* = 21). (*Inset*) Higher proportion of abnormal fibers (combined COX^−^/SDH^+^ and mitochondria-depleted fibers) in COPD patients (957 of 8136 fibers) compared to control subjects (158 of 5182 fibers). Graphs show mean ± SEM (***P < 0.001). Scale bar is 50 μm

To permit in situ characterization of fiber-to-fiber variation in myocellular oxidative capacity, dual COX and SDH histochemistry was performed and revealed a mosaic pattern of staining with COX-deficient and COX-normal fibers within the COPD patient muscle (Fig. 3a). We observed a greater presence of COX^−^/SDH^+^ fibers in COPD muscle; 8.0 ± 2.1 % versus 1.5 ± 0.4 % in controls; *P* < 0.03; Fig. 3b), consistent with the greater occurrence of mtDNA deletions observed in COPD muscle. Furthermore, we also observed a trend (*P* = 0.10) to greater abundance of fibers with very low levels of both COX and SDH activities (COX^low^/SDH^low^) in COPD muscle compared to age-matched controls (Fig. 3a, b; 7.5 ± 3.3 % in COPD vs. 1.4 ± 0.4 % in controls). Taken together, COPD patients had greater levels of respiratory-abnormal (COX^−^/SDH+ and COX^low^/SDH^low^ together) fibers than controls (14.4 ± 3.7 vs. 2.9 ± 0.8 %; *P* < 0.05).

### Depletion of mitochondrial DNA in COPD locomotor muscle

**Fig. 4:**
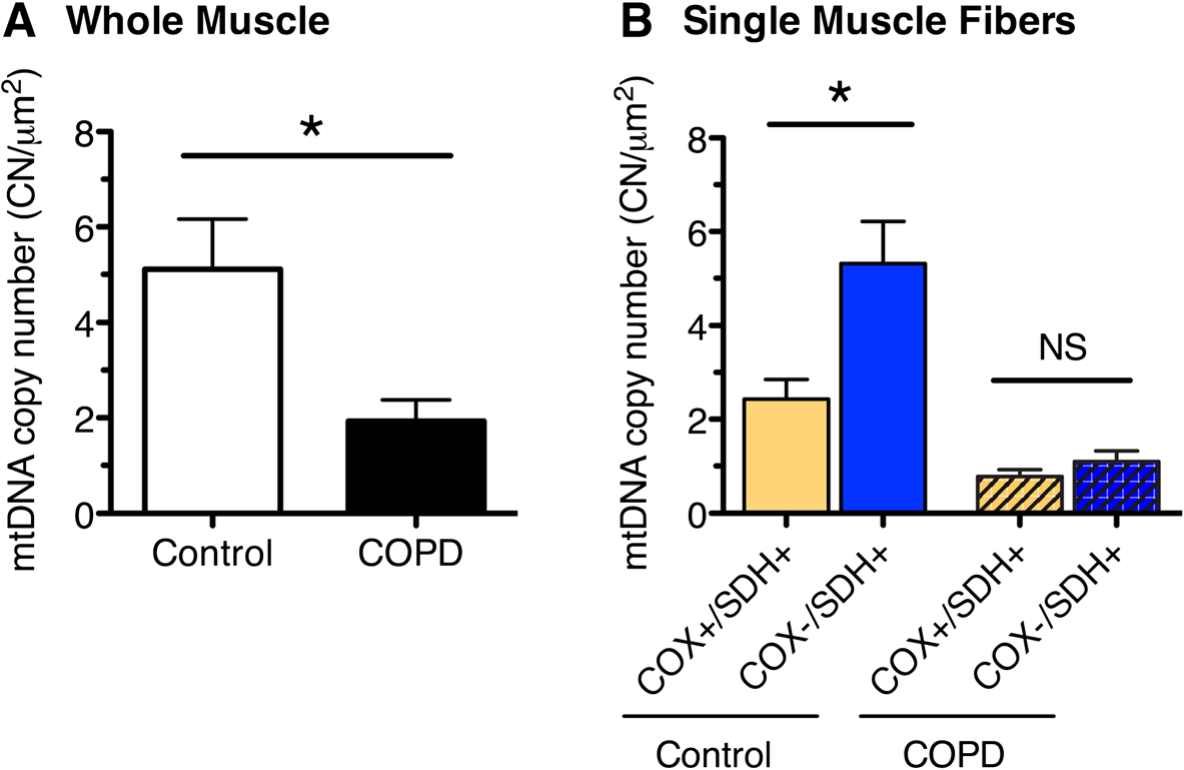
MtDNA copy number measured by real-time PCR in *vastus lateralis* muscle of control and COPD subjects. **a** Lower mtDNA copy number in homogenate muscle samples of COPD (1.9 ± 0.45, *n* = 15) compared to controls (5.1 ± 1.1, *n* = 13; *P < 0.05). **b** MtDNA copy number in single COX-normal (COX^+^/SDH^+^; *n* = 12 fibers per group) and COX-deficient (COX^−^/SDH^+^; *n* = 13 fibers per group) muscle fibers, demonstrating an upregulation of mtDNA copy number in COX-deficient fibers of controls (5.3 ± 0.89 compared to 2.4 ± 0.42 in COX-normal) but not in COPD muscle (1.1 ± 0.23 in COX-deficient fibers compared to 0.78 ± 0.14 in COX-normal). Graphs show mean ± SEM (*P < 0.05)

The number of mtDNA copies measured in muscle homogenates of 15 COPD patients and 13 age-matched controls was significantly lower (*P* < 0.05) in COPD (1.9 ± 0.45 copies per μm^2^) compared to controls (5.1 ± 1.1 copies per μm^2^; Fig. 4a). Within individual fibers that were laser captured (Fig. 4b), a two-way ANOVA (group × COX status) also revealed main effects for differences between COPD and controls, with lower copy number in COPD, and no interaction with COX status (copy number in COX-normal fibers: control 2.4 ± 0.42 vs. COPD 0.78 ± 0.14 copies per μm^2^, *P* < 0.05; copy number in COX^−^/SDH^+^ fibers: controls 5.3 ± 0.89, COPD 1.1 ± 0.23 copies per μm^2^, *P* < 0.05). Interestingly, whereas there was a higher copy number in COX^−^/SDH^+^ compared to COX-normal fibers in the control group (as occurs in other conditions exhibiting such as mtDNA disease and normal aging), this was not the case in COPD, revealing a failed compensatory mitochondrial biogenesis in COX^−^/SDH^+^ fibers of COPD patients. We were unable to extract mtDNA from the COX^low^/SDH^low^ fibers, suggesting they had a very low mtDNA copy number.

### Abnormal expression of markers of mitochondrial biogenesis in COPD locomotor muscle

**Fig. 5:**
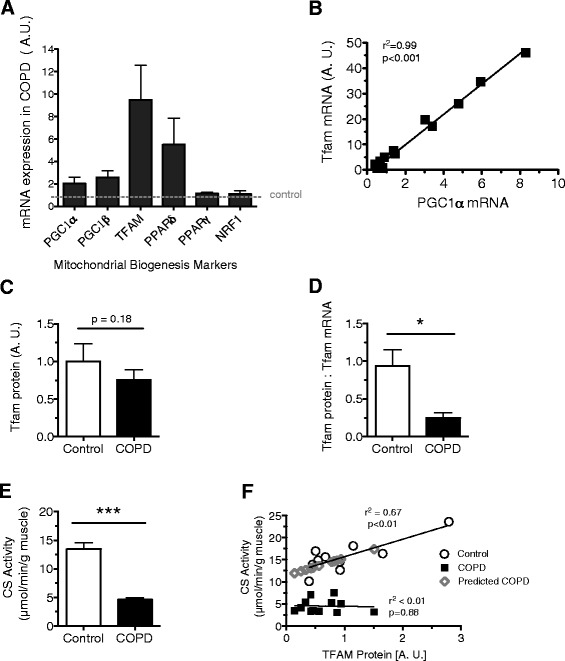
Markers of mitochondrial biogenesis and oxidative metabolism in *vastus lateralis* muscle homogenates of COPD patients relative to controls. **a** Higher levels of mitochondrial biogenesis and oxidative metabolism markers in COPD (two-way ANOVA revealed the significant main effect of disease status, *P* < 0.01; TFAM and PGC-1α; COPD *n* = 17, controls *n* = 11, NRF1, PGC1β, PPAR δ, and PPAR γ; COPD *n* = 9 controls *n* = 9). **b** Significant correlation between upregulated mitochondrial biogenesis signals PGC-1α and TFAM in COPD patients (*r* ^2^ = 0.99, *n* = 10; *P* < 0.001). **c** Levels of TFAM protein in COPD patients (0.76 ± 0.14, *n* = 20) and control subjects (1.0 ± 0.23, *n* = 10; *P* = 0.18) assessed by Western blot. **d** Lower ratio of TFAM protein relative to TFAM mRNA in COPD patients (0.25 ± 0.07) compared to controls (0.94 ± 0.2; *P < 0.5). **e** Lower levels of citric synthase activity in COPD patients (4.62 ± 0.33, *n* = 19) than controls (13.44 ± 1.14, *n* = 14; ***P < 0.001). **f** Positive relationship between TFAM and CS exists in controls (*r* ^2^ = 0.67, *P* < 0.1) but not in COPD patients (*r* ^2^ < 0.1). Measured levels of CS activity in COPD are 51 % lower than predicted for the measured TFAM protein. Graphs show mean ± SEM

The expression levels of important regulators of mitochondrial biogenesis and oxidative metabolism are shown in Fig. 5a. A two-way ANOVA revealed a significant main effect of subject group on levels of mRNA (*P* = 0.0073; Fig. 5a) with no interaction (*P* = 0.061) indicating that these regulators were increased in COPD. A significant correlation between PGC-1α and TFAM transcript levels was observed in COPD patients (*r*^2^ = 0.99, *n* = 10; *P* < 0.001), suggesting coordinated upregulation of mitochondrial biogenic factors in the patient group (Fig. 5b). Despite this coordinated regulation, the amount of TFAM protein (control 1.0 ± 0.23, COPD 0.76 ± 0.14; *P* = 0.18) did not differ between COPD and control subjects (Fig. 5c), and the ratio of TFAM protein to TFAM transcript was dramatically reduced in COPD patients (0.23 ± 0.06) compared to controls (0.94 ± 0.22; *P* < 0.05, Fig. 5d), with the latter observation suggesting impaired translation of TFAM transcript to protein in COPD muscle. Citrate synthase (CS) activity, a marker of mitochondrial content, was lower in COPD patients (4.6 ± 0.33 μmol per min per g muscle) than in controls (13.4 ± 1.1 μmol per min per g muscle, *P* < 0.001, Fig. 5e). Furthermore, a significant and positive correlation between TFAM and CS was found in controls (*r*^2^ = 0.67, *P* < 0.01, Fig. 5f), suggesting a coordinated regulation of nuclear and mitochondrial components of organelle biogenesis. However, there was no relationship between CS activity and TFAM protein in COPD patients (*r*^2^ < 0.1), where the measured levels of CS activity in COPD are 51 % lower than predicted for the measured TFAM protein. These latter observations suggest the TFAM protein itself may be defective in COPD.

### Abnormal TFAM expression in COX-deficient myofibers in COPD patients

**Fig. 6:**
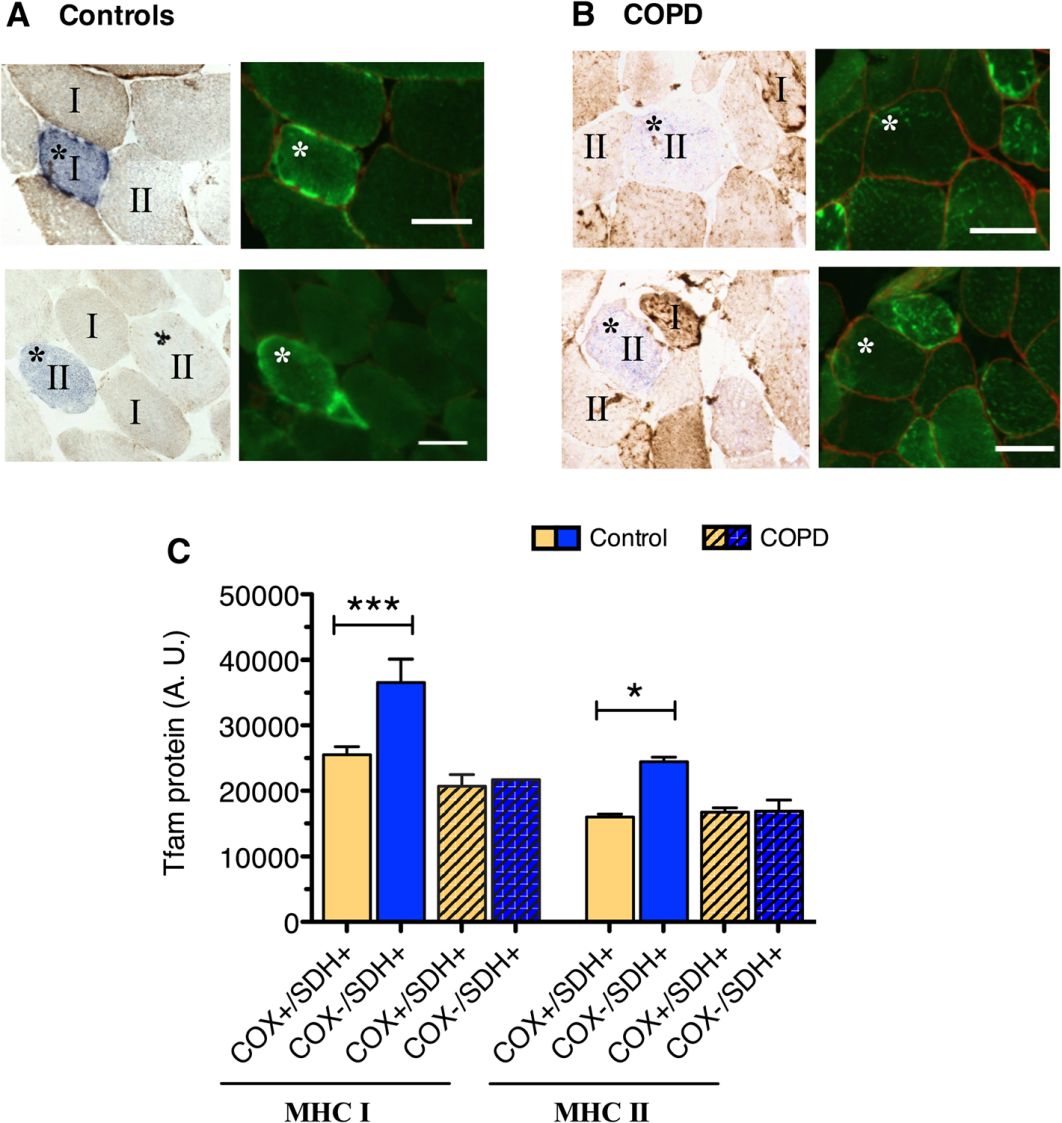
Abnormal expression pattern of TFAM protein in individual COX-deficient (COX^−^/SDH^+^) fibers of COPD locomotor muscle. **a–b** Representative images of muscle immunofluorescently labeled for TFAM showing higher levels of **a** TFAM protein in COX-deficient fibers compared to COX-normal fibers in *vastus lateralis* muscle of control subjects and **b** no difference in TFAM protein between COX-normal and COX-deficient fibers in COPD patients. Slow-twitch and fast-twitch muscle fibers identified as *I* and *II*, respectively, representing myosin heavy chain (MHC) I and MHC II fibers; scale bar is 50 μm. **c** Quantification of TFAM protein content in individual COX-normal (COX^+^/SDH^+^) and COX-deficient (COX^−^/SDH^+^) fibers reveals higher content in COX-deficient compared to COX-normal slow-twitch (***P < 0.001) and fast-twitch fibers (*P < 0.05) within control muscle, but no such difference in COPD muscle

Immunofluorescent labeling for TFAM protein in individual myofibers revealed an abnormal expression pattern in COPD locomotor muscle when compared to healthy controls. In control subjects, there was a dramatic increase in TFAM expression observed in COX-deficient myofibers, compared to COX-normal myofibers, consistent with the mtDNA copy number findings (Fig. 6a). In contrast, no differences in TFAM expression were detected between COX-deficient and COX-normal myofibers in COPD patients (Fig. 6b). In controls, this differential pattern of staining between COX-deficient and COX-normal fibers was seen for both slow MHC type I- and fast MHC type II-expressing fibers (type I COX-deficient fibers 36,553 ± 3575 A.U., COX-normal 25,510 ± 1241 A.U., *P* < 0.001; type II COX-deficient fibers 24,457 ± 673.2 A.U., COX-normal 16,007 ± 414.3, *P* < 0.05). In contrast, muscle fibers from COPD patients exhibited no differences in TFAM expression between COX-normal and COX-deficient fibers of neither MHC type (type 1 COX-deficient 21,706, COX-normal 20,688 ± 1782 A.U.; type 2 COX-deficient 16,868 ± 1737, COX-normal 16,731 ± 652.4; Fig. 6c) corresponding to a failed regulatory response across both slow and fast muscle fibers.

## Discussion

A low skeletal muscle oxidative capacity is a key feature of COPD, and this plays a key role in the severe exercise intolerance evident in this patient population. Despite significant study, the causes of low muscle oxidative capacity in COPD remain to be understood and consensus has not been reached on whether this impairment is simply a result of the very low physical activity levels seen in these patients. As such, the purpose of our study was to evaluate whether the high oxidative stress environment typical of COPD, wherein oxidative damage to muscle proteins and lipids is well established, also translates to DNA, with a specific objective of quantifying the impact on mtDNA and oxidative impairment in COPD locomotor muscle. Consistent with our hypothesis, we showed that COPD muscle exhibits higher levels of oxidatively damaged DNA and mtDNA deletions and that this was accompanied by a higher abundance of muscle fibers deficient in COX activity (a histological marker of high mtDNA mutation load [24, 46, 47]). Transcripts of mitochondrial biogenesis and oxidative metabolism were upregulated; however, this did not result in increased TFAM protein or mtDNA copy number. Furthermore, by exploiting measures at the single myocyte level, we were able to reveal that COPD patients exhibit an impaired mitochondrial biogenesis response in these respiratory-deficient fibers. This latter observation provides the first substantive evidence that low muscle oxidative capacity in COPD cannot be explained by very low physical activity levels alone and is likely driven by the COPD disease pathophysiology.

### Impact of oxidative stress on nuclear and mitochondrial DNA

Oxidative stress is well documented in COPD muscle, with oxidative damage to lipids and proteins having been measured most commonly [20, 48, 49]. Our results extend these findings to show for the first time that COPD muscle also exhibits higher levels of 8-OHdG, a commonly occurring oxidative lesion to DNA [40, 41]. These results are consistent with a recent study showing the mitochondrial genome is targeted by oxidative damage in COPD muscle [13], where a lower relative amplification of mtDNA was reported using an assay that is inhibited by oxidative DNA damage. Our study extends upon these findings by revealing, through long-range PCR, that mtDNA from COPD locomotor muscle is more prone to deletion mutations, reporting a greater frequency of COPD patients (72 %) exhibiting mtDNA deletions compared to the age-matched control group (16 %). The idea that this oxidative damage to DNA in general (8-OHdG) and mtDNA in particular (mtDNA deletions) is related to the high oxidative stress in COPD is supported by the observation that patients who had mtDNA deletions also had a higher 8-OHdG burden and more smoking pack-years than COPD patients without mtDNA deletions, noting that smoking has been previously shown to increase 8-OHdG [50]. We also observed that patients harboring mtDNA deletions had a lower VO_2max_, showing a greater disease impact on aerobic exercise capacity in affected patients.

### Consequences of mtDNA damage on cellular oxidative capacity

Previous studies in primary mitochondrial disease and aging have established that the presence of mtDNA mutations within a muscle cell disrupts the transcription of genes encoding mitochondrial subunits and can lead to a respiratory chain deficiency [23–25, 27]. The manifestation of a respiratory chain deficiency depends upon unique aspects of mitochondrial genetics. Specifically, because mitochondria contain multiple copies of mtDNA, when mtDNA mutations are present, they usually co-exist in the cell with intact wild-type mtDNA. In this setting, a cellular biochemical defect is expressed when the proportion of mutated mtDNA exceeds a critical threshold, and this is exacerbated when the absolute level of wild-type molecules is low [27, 29, 45]. Since mtDNA encodes three subunits of complex IV (COX), and none of complex II (SDH), a mutation in the form of a mtDNA deletion often results in abnormal COX activity, while SDH activity will remain unchanged or become hyperreactive [43]. Hence, a COX^−^/SDH^+^ fiber is a commonly used biomarker of respiratory chain deficiency secondary to high mtDNA mutation load in a variety of clinical conditions [24, 26, 46, 47]. The overall proportion of these COX-deficient fibers was more than five times higher (8 %) in our COPD patients than in healthy controls, who had levels (1.5 %) consistent with previous reports for the ages studied (up to 2 %) [26].

In addition to COX^−^/SDH^+^ fibers, we detected a marked presence of COX^low^/SDH^low^ fibers in COPD patients. These fibers, lacking activity of both OXPHOS complexes, have previously been described by Gosker and colleagues [51, 52] to exist in greater proportions in COPD muscle compared to controls. The presence of these fibers in healthy aging is not highly reported; however, their prevalence in COPD muscle would have functional implications on muscle oxidative capacity. If we combine COX^−^/SDH^+^ and COX^low^/SDH^low^ fibers in our COPD group, ~14 % of muscle fibers have negligible respiratory capacity compared to less than 3 % in controls. This level is clearly an important contributor to the overall reduced muscle oxidative capacity that has been established in COPD muscle as a whole. Further to these points, quantification of mtDNA copy number revealed a markedly reduced mtDNA copy number in both whole muscle homogenates and in laser captured single fibers (further elaborated upon below). These findings indicate a lower availability of templates for transcription and translation of the 13-mtDNA encoded respiratory chain subunits, which is consistent with the low muscle oxidative capacity in COPD. Future application of a newly developed quadruple immunofluorescent technique [53], allowing for the quantification of key respiratory chain subunits of complex I and IV, to muscle from COPD patients may likely expose impairment in multiple mitochondrial encoded ETC complexes.

### Implications of single fiber experiments in COPD muscle

A crucial question is whether the reported lower mtDNA copy number reflects a pathological process of COPD or is a consequence of a reduced drive on mitochondrial biogenesis associated with the sedentary lifestyle typical of these patients. Controversy regarding the exact contribution of a sedentary lifestyle to the low muscle oxidative capacity in COPD has persisted [2, 54] because recent reports have not been able to demonstrate an association between physical activity levels and a reduced muscle oxidative phenotype [55, 56]. Moreover, a low muscle oxidative capacity is seen in relatively mild COPD patients [5] who do not exhibit the same degree of sedentary lifestyle as more severely affected patients [18, 57]. Clearly, further study is warranted using novel approaches to this question. In view of the heterogeneity of impact from one muscle fiber to another in the context of both normal aging and mitochondrial disease, it seems likely that single fiber approaches to the problem in COPD muscle may yield a clearer understanding.

In previous studies in primary mitochondrial disease patients, mtDNA copy number and TFAM protein levels were shown to be markedly upregulated in fibers with severe oxidative impairment characterized by COX deficiency [28, 29], demonstrating a strong drive on mitochondrial biogenesis in these oxidatively impaired fibers. COX deficiency also occurs in some fibers with normal aging, and these fibers also exhibit upregulated mtDNA copy number [58]. Taken together, these findings reflect a normal compensatory response to the severe energy deficit in fibers with high mtDNA mutation loads, in a futile attempt to maintain adequate ATP levels for normal cellular function [29]. Thus, in our current experiments, we exploited this phenomenon as a novel way of revealing whether there is an impaired capacity for mitochondrial biogenesis in COPD muscle independent of their sedentary lifestyle. Specifically, we reasoned that if mitochondrial biogenesis is impaired in COPD muscle, COX^−^/SDH^+^ fibers in these patients should demonstrate a limited capacity for increasing mtDNA copy number relative to what is seen in COX^−^/SDH^+^ fibers of healthy age-matched controls. Note that despite similarly low VO_2max_ (~30–50 % of age-matched predicted values) and physical activity levels (<6000 steps per day) between patients with mtDNA deletions [31, 59] and COPD patients [56, 57], a robust upregulation of mtDNA copy number and mitochondrial biogenesis is seen in the oxidative-impaired (COX^−^/SDH^+^) fibers relative to normal fibers in mitochondrial disease patients. Consistent with this reasoning, not only was there a lack of upregulation in mtDNA copy number in these oxidatively impaired fibers of our COPD disease patients but the COX^−^/SDH^+^ phenotype differed from that which is seen in primary mitochondrial disease where there is a hyperreactivity of SDH staining reflecting the compensatory attempt to overcome the energy deficit in these fibers. Indeed, we were not able to detect any SDH hyperreactivity in COX^−^/SDH^+^ fibers of our COPD muscle, further supporting the notion of failed mitochondrial biogenesis. Thus, the fact that this response is impaired in the oxidative-impaired fibers in COPD locomotor muscle argues strongly that this is a pathological alteration that is not related to their very sedentary lifestyle.

Whereas it has been previously suggested that the mitochondrial biogenesis response in COPD muscle under basal conditions is suppressed based upon low PGC-1α and TFAM [14], other studies suggest no impairment in mitochondrial biogenesis based upon a normal PGC-1α transcript response to acute exercise [13, 60]. Notwithstanding these apparent contradictions, our new results clearly demonstrate a deficit in mitochondrial biogenesis in COPD muscle but that this occurs downstream of PGC-1α and TFAM transcripts at the level of TFAM protein. Specifically, in stark contrast to the healthy controls, we observed a lack of upregulation in TFAM protein within the COX^−^/SDH^+^ fibers of COPD patients, and this was independent of fiber type (Fig. 6c). Thus, whereas our data support some aspects of the previous suggestion of a normal mitochondrial biogenesis response in COPD muscle by showing the coupling between PGC-1α and TFAM transcription is maintained, in sharp contrast to a normal response, we also show a marked uncoupling between TFAM transcript levels and TFAM protein, suggesting a problem in TFAM translation in COPD muscle. Further to this point, whereas we observed the expected stoichiometry between nuclear and mitochondrial components of the mitochondrial biogenesis pathway in age-matched controls (based upon a close relationship between the nuclear encoded citrate synthase enzyme activity and TFAM protein levels), there was no relationship between these components in COPD, suggesting that even the limited TFAM protein that is available in COPD muscle may be compromised in its function. While future studies are needed to confirm altered TFAM function in COPD muscle, it is known that protein oxidation can modify DNA binding of transcription factors and make them more prone to proteolytic degradation [61]. Therefore, direct assessment of TFAM’s ability to initiate mtDNA transcription and replication (as performed by Karamanlidis and colleagues [62]) is among the next important steps in understanding why mtDNA copy number and oxidative capacity are low in COPD muscle.

Interestingly, in human heart failure, another disease associated with high oxidative stress, high levels of 8-OHdG have been associated with low levels of TFAM protein and impaired mtDNA replication, with the resultant mitochondrial depletion shown to be independent of PGC-1α levels [62]. This is strikingly similar to what we observed in our COPD patient muscle and strongly implicates a common mechanism related to oxidative stress. Additionally, inflammation, an upstream mediator of oxidative stress, has also been linked to mitochondrial abnormalities in various clinical conditions, with a recent study reporting high levels of COX^−^/SDH^+^ fibers carrying mtDNA deletions in muscle of patients with sporadic inclusion body myositis, a progressive myopathy characterized by excessive inflammation [47]. Moreover, in inclusion body myositis patient muscle, a correlation between the level of COX^−^/SDH^+^ fibers and the degree of T lymphocyte infiltrate was detected, leading the authors to speculate that the chronic inflammatory environment is important in driving the mitochondrial abnormalities detected in this population. As such, the evidence is accumulating that chronic inflammation, oxidative stress, and mitochondrial abnormalities are linked, and further investigation into the mechanism underlying the impairment in mtDNA replicative capacity in COPD is certainly warranted.

## Conclusions

Our current results add to previous studies documenting the impact of oxidative damage in COPD locomotor muscle by showing that COPD muscle exhibits higher levels of oxidatively damaged DNA and mtDNA deletions. Furthermore, COPD muscle exhibited an exacerbation of the normal age-related accumulation of COX^−^/SDH^+^ muscle fibers (a histological marker of high mtDNA mutation load). Following up on this observation using novel techniques first developed for the study of the cellular consequences of mtDNA mutations in primary mitochondrial disease, we showed for the first time that there is an impaired mitochondrial biogenesis response in these respiratory-deficient fibers in COPD muscle. The significance of this observation is that it provides compelling evidence that low muscle oxidative capacity in COPD is driven by the COPD disease pathophysiology rather than merely being the consequence of their very sedentary behavior.

We propose that the impairment in mitochondrial biogenesis revealed in our study, which manifest as a compromised capacity for mtDNA replication in oxidatively impaired fibers, leads to a gradual erosion of muscle oxidative capacity in COPD. Furthermore, this impaired capacity for mtDNA replication results in a chronically low mtDNA copy number that in turn renders myocytes more vulnerable to further mtDNA damage-induced oxidative depletion in COPD (because fewer mtDNA copies mean a faster attainment of the cellular deletion threshold for phenotypic manifestation of the oxidative impairment) and further exacerbates the muscle oxidative deficit and contributes to impaired fatigue resistance in COPD patients.

## Abbreviations

8-OHdG: 8-hydroxy-2-deoxyguanosine
BMI: body mass index
COPD: chronic obstructive pulmonary disease
COX: cytochrome *c* oxidase
CS: citrate synthase
DAB: diaminobenzidine tetrahydrochloride
DLCO: lung diffusion capacity
FEV_1_: forced expiratory volume in one second
FVC: forced vital capacity
HNE: 4-hydroxynonenal
MHC: myosin heavy chain
mtDNA: mitochondrial DNA
MTND1: mitochondrially encoded NADH dehydrogenase 1 region
TFAM: mitochondrial transcription factor A
PGC-1α: peroxisome proliferator-activated receptor γ coactivator 1α
RV: residual volume
SDH: succinate dehydrogenase
TLC: total lung capacity
VO_2_: peak rate of oxygen consumption
